# Stabilizing Effect of Soluplus on Erlotinib Metastable Crystal Form in Microparticles and Amorphous Solid Dispersions

**DOI:** 10.3390/polym14061241

**Published:** 2022-03-19

**Authors:** Shuyu Jia, Shangqi Ning, Yuting Leng, Qiufang Jing, Zhongyu Xu, Fuzheng Ren

**Affiliations:** Shanghai Frontiers Science Center of Optogenetic Techniques for Cell Metabolism, Engineering Research Center of Pharmaceutical Process Chemistry, Ministry of Education, Shanghai Key Laboratory of New Drug Design, School of Pharmacy, East China University of Science and Technology, Shanghai 200237, China; y30201155@mail.ecust.edu.cn (S.J.); y30171066@mail.ecust.edu.cn (S.N.); y45190565@mail.ecust.edu.cn (Y.L.); qfjing@ecust.edu.cn (Q.J.); zyxu@ecust.edu.cn (Z.X.)

**Keywords:** erlotinib, soluplus, microparticles, solid dispersions, stabilization

## Abstract

Microparticles (MPs) and amorphous solid dispersions (SDs) are effective methods to improve the dissolution of insoluble drugs. However, stability is a concern for these two high-energy systems, resulting from high surface area and amorphous polymorph, respectively. As an amphiphilic polymer, Soluplus (SOL) is usually used as a carrier in SDs. In this study, erlotinib microparticles (ERL MPs) and erlotinib solid dispersions (ERL SDs) were prepared with SOL by bottom-up technology and solvent evaporation. The solid-state properties of ERL MPs and ERL SDs were characterized by Differential Scanning Calorimetry (DSC), Powder X-Ray Diffraction (PXRD) and Scanning Electron Microscopy (SEM). The ERL MPs existed in a metastable crystal form A while the ERL SDs existed in an amorphous state. Fourier transform infrared spectroscopy (FT-IR) showed that there was a hydrogen bond interaction between the N-H group of ERL and the carbonyl group of SOL in ERL MPs and SDs. The dissolution profiles of ERL SDs and ERL MPs were improved significantly. ERL MPs showed better stability than ERL SDs in accelerated stability test. The discrepant stabilizing effects of polymer SOL in two systems may provide effective ideas for solubilization of insoluble drugs and the stability of drugs after recrystallization.

## 1. Introduction

Since more than 40% of commercial drugs suffer from poor bioavailability and low therapeutic efficacy due to low aqueous solubility [1], it is essential to find effective methods to improve aqueous solubility of poorly water-soluble drugs. Various strategies, including employment of surfactants, formation of solid dispersions (SDs) and reduction of particle size, have been widely used to overcome drug solubility problems [2,3,4].

Drugs are generally in an amorphous state in SDs and uniformly dispersed in hydrophilic polymers, which increases in transient drug solubility and thus oral bioavailability [5,6]. However, along with the improved solubility comes the reduction of physical stability of the drug, which refers to recrystallization or phase separation during storage. Compared with crystalline form, the amorphous form has higher energy which is thermodynamically unstable and readily recrystallizes to a more stable crystalline state [7]. Furthermore, the drug has a tendency to recrystallize from supersaturated solutions generated by SDs, which may affect drug absorption [8]. In order to improve the stability of SDs, multi-phase mixed carriers have been used in recent years. The study by Fan W. et al. showed that the solubility and dissolution of poorly water-soluble drugs can be improved by preparing ternary amorphous SDs, and the stability can be maintained for a long time [9]. Additionally, Bharate S. S. prepared the binary SD formulation, displaying high aqueous solubility and superior dissolution performance compared to the parent molecule [10].

Another method to improve aqueous solubility of poorly water-soluble drugs is to reduce the particle size to micron or nanometer level. Only some products need to be prepared into nanoparticles (NPs) to enhance therapeutic efficacy, such as Rapamune^®®^, Emend^®®^ and Tricor^®®^, approved by the FDA for marketing [11]. Most drugs can achieve high bioavailability as microparticles (MPs).

According to the preparation process, the methods to reduce particle size include “top-down” and “bottom-up” technologies. The “top-down” approach breaks large bulk particles down by milling or high-pressure homogenization [12]. While the products on the market mainly use “top-down” technology, this approach is a time-consuming and energy-consuming process. In addition, it is still difficult to narrow particle size distributions and avoid the risk of contaminating the product by the attrition of milling media [13]. Based on precipitation of drug from a supersaturated solution [14], the bottom-up techniques have advantages that include low energy consumption and simple instrumentation. Therefore, bottom-up techniques have a great potential for dissolution enhancement of water-insoluble drugs [15]. However, bottom-up technology is difficult to scale up, and the particle size of the MPs varies significantly with the experimental parameters [16]. Fortunately, the solid state of the crystalline drugs can be well controlled in the presence of polymers as stabilizers during the precipitation process [17].

Polymers, which can act as stabilizers in both SDs and MPs, are increasingly popular in solubility and dissolution enhancement of poorly water-soluble drugs. While a consensus has not been reached concerning the stabilizing mechanism of polymers, various studies have confirmed the significant role played by supramolecular interactions such as hydrophobic interactions, van der Waals forces and hydrogen bonding [18]. Polymers can prevent drug nucleation and crystallization by interrupting long range ordered structure formed among drug molecules through hydrogen bonds or decreasing the mobility of drug molecules [19,20]. In addition, polymers can inhibit hydrophobic aggregation of drug crystals through absorption onto surface of the particles [21]. However, the question about how to select an appropriate stabilizer for a certain drug remains open due to inherent properties of different drugs.

Amphiphilic polymers have been found to have advantages over traditional polymers for improving drug dissolution and absorption [22]. Soluplus (SOL), polyvinyl caprolactam-polyvinyl acetate-polyethylene glycol graft copolymer, is an amphiphilic polymer mainly used in solid dispersions. It not only displays superior solubilizing effects for Biopharmaceutics Classification System (BCS) class II drugs, but also forms micelles in aqueous solution, exhibiting better solubilization ability than conventional surfactants [23]. In addition, its surface activity is beneficial for maintaining the supersaturation of poorly soluble drugs in the gastrointestinal (GI) tract [24].

Erlotinib (ERL), an inhibitor of the epidermal growth-factor receptor (EGFR) tyrosine kinase, was approved by the US Food and Drug Administration (FDA) for the treatment of local or advanced metastatic non-small cell lung cancer and pancreatic cancer in 2004 [25]. However, it is classified as a BCS class II drug due to its low aqueous solubility and high permeability [26], which may be a key reason explaining its low bioavailability. Therefore, it is necessary to improve its solubility and dissolution by formulation strategies.

This study primarily attempted to investigate the stabilizing effect of SOL on drug in two binary systems, SDs and MPs. ERL MPs and ERL SDs were prepared using SOL as carrier. Solid characterizations were performed by scanning electronic microscopy (SEM), differential scanning calorimetry (DSC), thermogravimetric analysis (TGA), powder X-ray diffraction (PXRD) and Fourier transform infrared spectroscopy (FT-IR). Meanwhile, dissolution and stability studies were conducted. The role of polymer SOL in different systems provided new insights into the solubilization of insoluble drugs and the stability of drugs after recrystallization.

## 2. Materials and Methods

### 2.1. Materials

**Figure 1:**
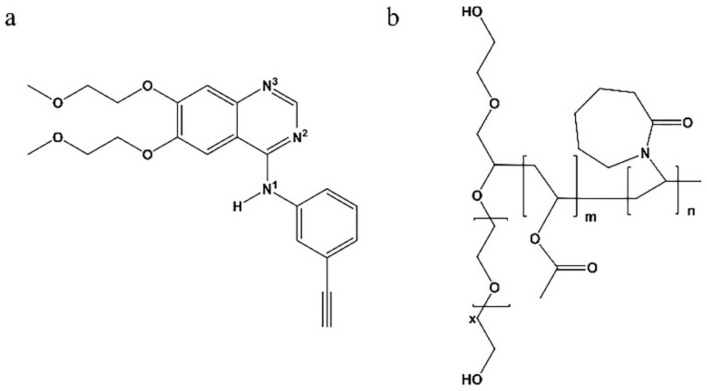
The molecular structures of (**a**) ERL and (**b**) SOL.

ERL (C_22_H_23_N_3_O_4_) was purchased from Adamas Reagent Co., Ltd. (Shanghai, China). SOL was supplied by BASF Co., Ltd. (Shanghai, China). Dimethyl sulfoxide (DMSO) was purchased from Shanghai Aladdin Biochemical Technology Co., Ltd. (Shanghai, China). All other reagents and chemicals used in this study were of analytical grade. Figure 1 shows the chemical structures of ERL (Figure 1a) and SOL (Figure 1b).

### 2.2. Methods

#### 2.2.1. Preparation of ERL SDs

SDs of ERL and SOL at 1:3 (*w*/*w*) were prepared by solvent evaporation; 1 g ERL and 3 g SOL were dissolved in 300 mL of 1:1 (*v*/*v*) mixture of dichloromethane and methanol to form a uniform solution. Solvent removal was achieved by rotary evaporation in a water bath maintained at 40 °C. The ERL SDs were subsequently dried overnight in a vacuum oven at 40 °C, −0.1 M Pa to remove residual solvent. Then the powder was passed through an 80 mesh sieve and collected in a dryer for later use.

#### 2.2.2. Preparation of ERL MPs

MPs of ERL and SOL at 1:3 (*w*/*w*) were prepared by anti-solvent precipitation; 3 g SOL was dissolved in 300 mL water to obtain a clear aqueous solution; 1 g ERL was dissolved in 3 mL DMSO to obtain an organic solution. The organic solution was quickly introduced into SOL aqueous solution under mechanical stirring (500 rpm) at 25 °C. After 30 min, the obtained suspension was filtered through a 0.45 μm microporous membrane and the filter cake was washed with 20 mL water. The solid was collected and then dried in a vacuum oven overnight at 40 °C, −0.1 M Pa. ERL Control was prepared as described above without the addition of SOL to the aqueous solution.

#### 2.2.3. Preparation of ERL PMs

Physical mixtures (PMs) of ERL and SOL at 1:3 (*w*/*w*) were prepared using mortar and pestle by gentle grinding.

#### 2.2.4. Solubility

Solubility of ERL in water and SOL solution were determined by dispersing an excess amount of ERL in 100 mL water or SOL aqueous solution (10 mg/mL). The solutions were shaken at 200 rpm and 25 °C for 48 h in a shaking bath. The solutions were subsequently filtered through a 0.45 µm membrane and analyzed by HPLC at 335 nm (Agilent 1260) with a C18-reversed phase Diamonsil column (200 × 4.6 mm, 5 μm, Dikma Technologies, Beijing, China) maintained at 30 °C. The mobile phase consisted of 0.02 M aqueous phosphoric acid and acetonitrile (60:40). The flow rate of the mobile phase was maintained at 1 mL/min and the injection volume was 20 µL.

#### 2.2.5. Focused Beam Reflectance Measurement (FBRM)

Crystallization behavior of ERL in the absence and presence of SOL was investigated utilizing FBRM (G400, Mettler Toledo, Columbus, OH, USA) at 2 s sampling intervals. A solution of ERL (100 mg) in DMSO (1 mL) was rapidly injected into 30 mL SOL solution (10 mg/mL) maintained in EasyMax at 25 °C under mechanical stirring (500 rpm) with the FBRM probe inserted. The online data were collected until a stable count was observed.

#### 2.2.6. Particle Size Measurement

Particle size analyses of ERL MPs and ERL control were carried out using a laser particle size analyzer (Mastersizer 2000, Malvern Instruments, Malvern, UK) with drug saturated solution as dispersant to prevent small drug particles from dissolving.

#### 2.2.7. Powder X-ray Diffraction (PXRD)

The PXRD patterns of ERL, PMs, ERL control, ERL MPs, ERL SDs and SOL were recorded on an Ultima IV (Rigaku, Tokyo, Japan) using Cu Kα radiation (40 kV, 40 mA). The 2θ angle was from 3° to 45°. The scanning rate was 20°/min.

#### 2.2.8. Scanning Electronic Microscopy (SEM)

The surface morphologies of ERL, ERL SDs, ERL control and ERL MPs were captured by SEM (JSM-6360LV, JEOL, Tokyo, Japan) at an accelerating voltage of 15 kV. Samples were fixed on aluminum stubs and then coated with a gold layer prior to SEM analysis.

#### 2.2.9. Thermogravimetric Analysis (TGA)

The relationship between quality and temperature of ERL MPs and ERL control were performed on TGA (Universal Analysis 2000, TA Instruments, New Castle, DE, USA). Samples were prepared by placing 3–10 mg of material in an aluminum pan. It was then heated from ambient temperature to 350 °C with a heating rate of 10 °C/min. Nitrogen gas was used for purging with a flow rate of 50 mL/min.

#### 2.2.10. Differential Scanning Calorimetry (DSC)

The thermal properties of solids were recorded by DSC (Universal Analysis 2000, TA Instruments, New Castle, DE, USA). Samples (2–4 mg) were weighed and sealed in an aluminum pan and heated from 30 °C to 200 °C (ramp rate: 10 °C/min; flow rate: 50 mL/min (nitrogen)).

#### 2.2.11. Fourier Transform Infrared Spectroscopy (FT-IR)

To investigate interactions between ERL and SOL, PMs, ERL SDs and ERL MPs were analyzed on an FT-IR spectrophotometer (Cary 630; Agilent Technologies, Cary, NC, USA) in attenuated total reflectance mode. Spectra were recorded over the range of 4000–400 cm^−1^, with 64 parallel scans (at a resolution of 4 cm^−1^).

#### 2.2.12. Dissolution

The in vitro dissolution experiments were performed at 37 ± 0.5 °C with an RCZ-8M dissolution tester (TDTF, Tianjin, China) using the paddle method at a speed of 50 rpm. The tests were carried out in 200 mL phosphate buffer solution (pH 6.8) with 0.5% sodium dodecyl sulfate. Samples (5 mL) were collected at 5, 10, 20, 30, 45, 60, 90 and 120 min and filtered through a 0.45 μm microfiltration membrane (Titan Technology Co., Ltd., Shanghai, China). The filtrates were analyzed by the above HPLC method.

#### 2.2.13. Accelerated Stability Test

ERL SDs and ERL MPs were placed in aluminum packing and stored in a stability chamber at 40 °C and relative humidity of 75%. Samples were characterized after 1, 2, 3 and 6 months.

## 3. Results and Discussion

### 3.1. FBRM

**Figure 2:**
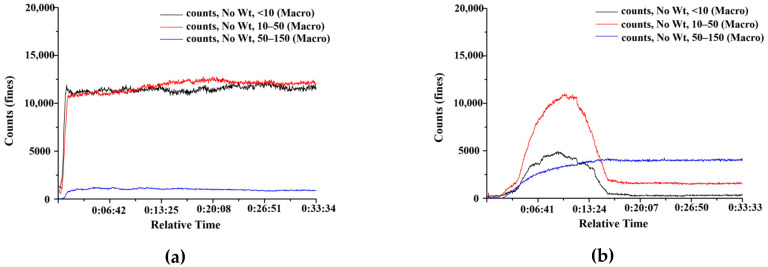
The variation curve of chord length of ERL MPs in (**a**) Water and (**b**) SOL solutions.

The crystallization processes of ERL in water and SOL aqueous solution were investigated using FBRM. Figure 2 shows the particle count variation of different chord lengths within 30 min. It can be seen that, upon injection of ERL solution into water (Figure 2a), a sharp and immediate increase in particle count for <10 µm was observed along with an increase in particle counts for 10–50 µm and 50–150 µm. After about 1 min, the particle counts stabilized and remained constant during the observation time. Upon injection of ERL solution in SOL solution (Figure 2b), an increase in particle count for 10–50 µm was observed along with increases in particle count for <10 µm and 50–150 µm. Generally, the increases were slower compared with those in water. According to solubility results of ERL determined previously, saturation solubility of ERL in water and SOL aqueous solution were 0.36 and 21.76 µg/mL, respectively. The solubilizing effect of SOL is believed to reduce the supersaturation of ERL, resulting in prolongation of the nucleation induction period. The particle count for chord lengths of 50–150 µm showed a slow increase between 2 min and 15 min. The particle count for chord lengths <10 µm and 10–50 µm began to decline after about 10 min. At 15 min, the particle counts for all chord lengths stabilized and remained constant for the rest of the experiment. The absence of any increase in particle count of all chord lengths indicated that the crystal growth was inhibited [27,28]. The experimental result showed that the SOL can be used as a crystal growth inhibitor. The FBRM analysis illustrated the inhibitory effect of SOL on ERL crystallization at a particle level, suggesting that SOL might act as a good stabilizer in ERL SDs and MPs.

### 3.2. Particle Size of ERL MPs

**Figure 3:**
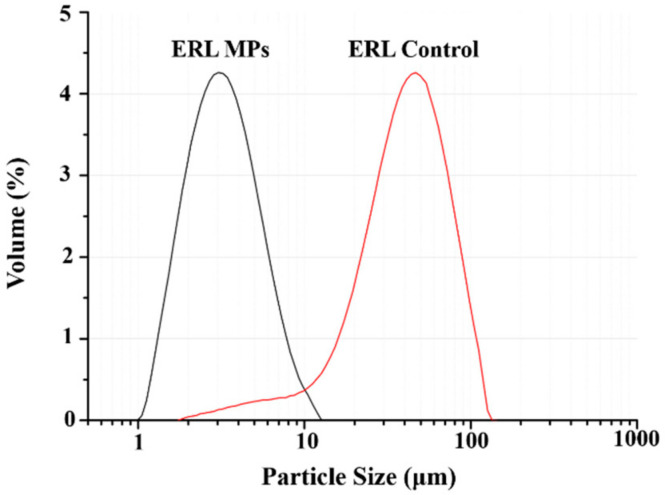
Particle size distribution of ERL MPs and ERL Control.

As shown in Figure 3, the particle size of ERL control was mainly distributed in the range of 10–100 μm, and D50 was 42.4 μm. ERL control was obtained by antisolvent crystallization. After the drug solution came in contact with the antisolvent, it would burst into nucleation. The supersaturation would decrease rapidly, which resulted in some small particles (<10 µm) in ERL Control. However, most of the particles were >10 µm and the particle size could not be well controlled. However, the particle size of ERL MPs was mainly in the range of 1–10 μm (D50 = 3.5μm), which was much smaller than ERL Control, corresponding to the observation in SEM. The addition of SOL significantly decreased the particle size of ERL, indicating that crystallization of ERL crystals could be effectively inhibited by SOL.

### 3.3. PXRD

**Figure 4:**
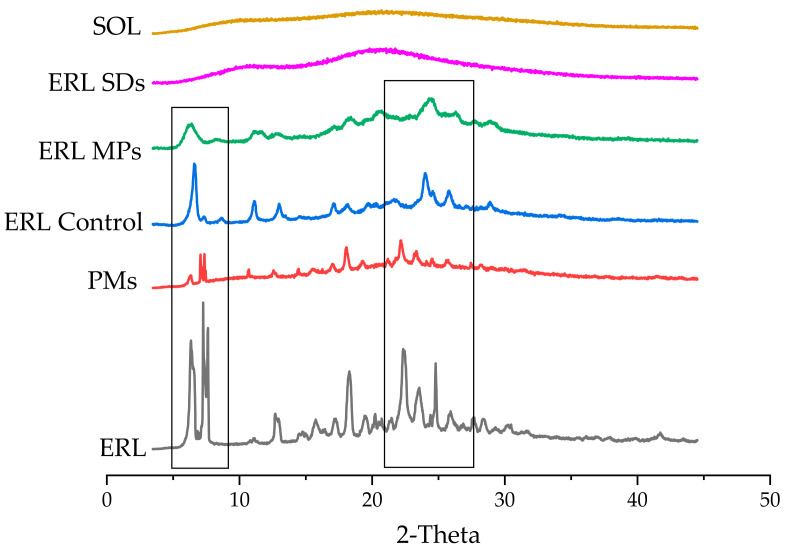
PXRD patterns of ERL, PMs, ERL control, ERL MPs, ERL SDs and SOL.

**Figure A1:**
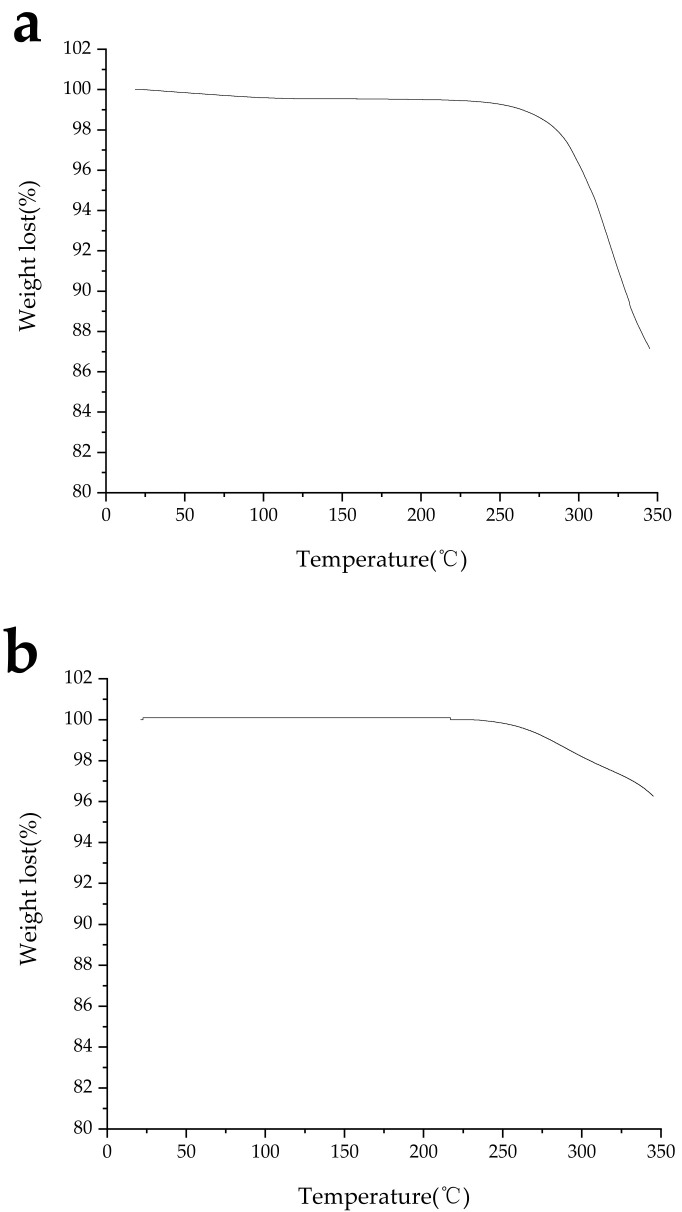
The TGA thermograms of (**a**) ERL MPs and (**b**) ERL Control.

The PXRD patterns of ERL, PMs, ERL control, ERL MPs, ERL SDs and SOL are shown in Figure 4. The ERL pattern showed strong and sharp diffraction peaks at 6.28°, 7.5°, 18.39°, 22.43°, 23.47° and 24.90° (2θ), revealing that it was in crystalline form B [29,30]. The SOL pattern contained no diffraction peaks, indicating its amorphous nature. However, there were no diffraction peaks in the pattern for the SDs generated from ERL and SOL, demonstrating that ERL was dispersed in the matrix in a completely amorphous form. ERL Control showed characteristic peaks at 6.7°, 11.06°, 12.94°, 23.98°, 24.61° and 28.78° (2θ), revealing a crystal transformation from form B to metastable form A [31], which was due to the anti-solvent crystallization process. ERL MPs formed by ERL and SOL also contained metastable form A, but crystallinity was decreased compared with ERL control, also confirming inhibition of ERL crystallization by SOL. According to studies by Shridhar H. et al., erlotinib was found to occur as two polymorphs and two hydrates depending on the crystallization conditions [32]. In order to clarify whether ERL MPs and ERL control were hydrates, TGA tests were performed (Figure A1). TGA showed that ERL MPs and ERL control had no weight loss peaks, indicating that form A was not monohydrate or trihydrate.

### 3.4. SEM

To explore whether or not SOL’s inhibitory effect could come into play in ERL preparations, ERL SDs and MPs were fabricated with SOL.

**Figure 5:**
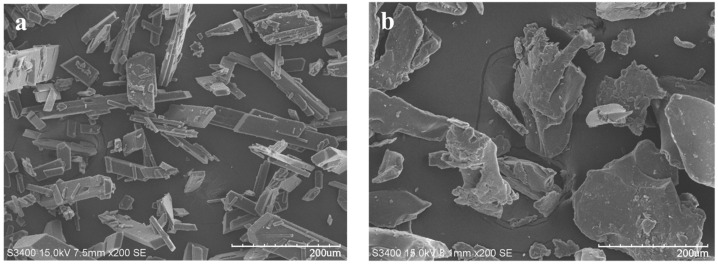

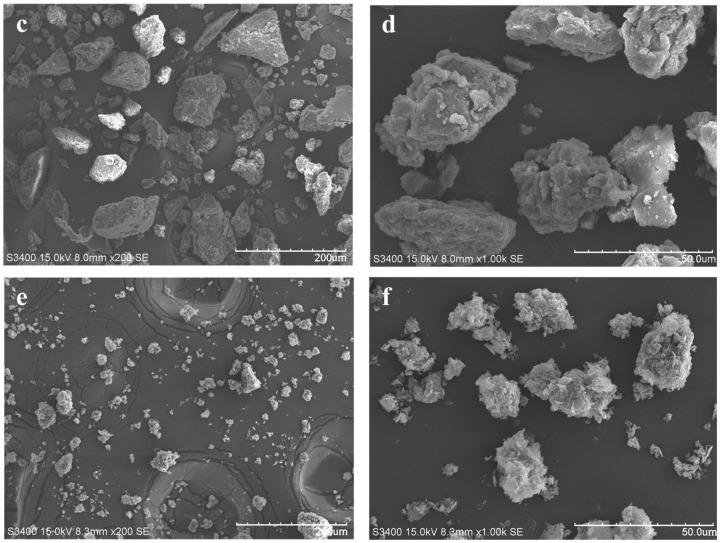
SEM images of (**a**) ERL, (**b**) ERL SDs, (**c**,**d**) ERL Control, (**e**,**f**) ERL MPs.

The surface morphologies of ERL, ERL SDs, ERL control and ERL MPs captured by SEM are presented in Figure 5. ERL crystals exhibited columnar habits with a smooth surface, lacking uniformity in size (Figure 5a). In the SEM of ERL SDs, the amorphous particles with irregular size exhibited a stratified structure, which might be due to solvent evaporation (Figure 5b). ERL control (Figure 5c,d) displayed a block-like crystalline structure with a rough surface. This morphological transition from columnar to block-like shape might indicate crystal form conversion. In ERL MPs (Figure 5e,f), the crystal size was smaller and more homogeneous than ERL control, suggesting that the addition of SOL reduced the ERL particle size. The small size and rough surface of MPs might contribute to improved dissolution of ERL. The drug content of ERL SDs and ERL MPs was measured due to the difference in surface morphologies. The drug content of ERL SDs was 25.13%, basically maintained at the theoretical value. However, the drug content of ERL MPs reached 59.06%, since SOL is a water-soluble polymer and was removed partly by washing during filtration.

### 3.5. DSC

**Figure 6:**
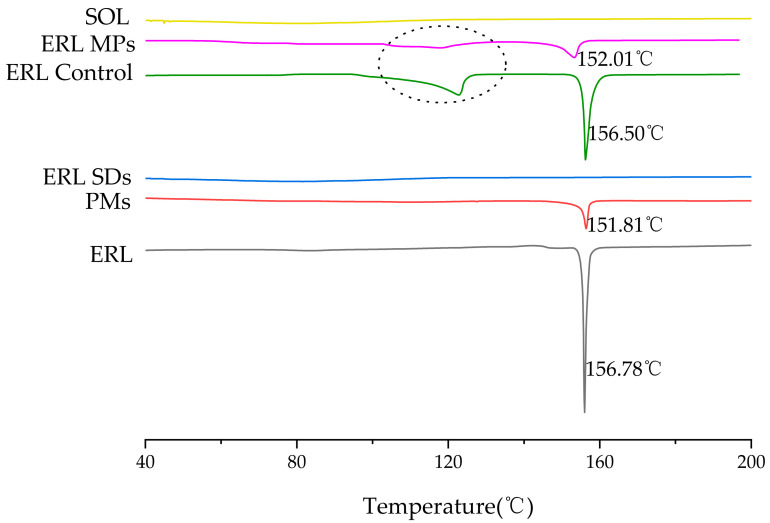
DSC curves of ERL, PMs, ERL SDs, ERL control, ERL MPs and SOL.

The DSC patterns of ERL, PMs, ERL SDs, ERL control and ERL MPs are shown in Figure 6. A sharp peak at 156.78 °C in the DSC of ERL corresponded to the melting point of form B. However, there were no peaks observed in the DSC of SOL, indicating its amorphous nature. There were no peaks observed in the DSC of ERL SDs. Typically, disappearance of a melting peak is indicative of a homogeneous amorphous system in which drug and polymer are dispersed uniformly [33]. Therefore, ERL was amorphously dispersed in the SOL matrix. In ERL control and ERL MPs, the second endothermic peak at a lower temperature was noted, which could be caused by the transformation of crystal form A to form B. Furthermore, the reduced melting peak in ERL MPs supported the crystallization inhibitory effect of SOL.

### 3.6. FT-IR

**Figure 7:**
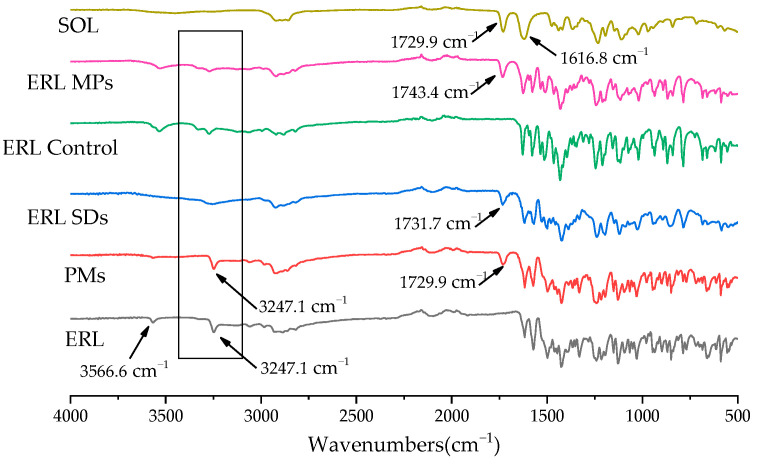
FT-IR spectra of ERL, PMs, ERL SDs, ERL control, ERL MPs and SOL from 4000 to 400 cm^−1^.

Figure 1 shows the chemical structures of ERL and SOL, where hydrogen-bond donors and acceptors can be seen, providing the structural basis for hydrogen-bonding interactions [34]. FT-IR was therefore utilized to study possible hydrogen-bonding interactions between ERL and SOL. The FT-IR spectra of ERL, PMs, ERL SDs, ERL control, ERL MPs and SOL are shown in Figure 7. The characteristic peak at 3247.1 cm^−1^ was assigned to the stretching vibration of the N-H moiety of ERL, and the peak at 3567.6 cm^−1^ was assigned to the stretching vibration of the terminal alkyne. In the spectrum of SOL, peaks detected at 1729.9 and 1616.8 cm^−1^ were attributed to the carbonyl groups of the ester and caprolactam ring, respectively [35]. In the spectrum of the PMs, peaks at 3247.1 cm^−1^ (N-H) and 1729.9 cm^−1^ (caprolactam ring) were a little weakened.

Usually, the shift of vibration peak position is due to crystal transformation or intermolecular hydrogen bonding [36]. After formation of the ERL SDs, the peak at 3247.1 cm^−1^ almost disappeared and the peak at 1729.9 cm^−1^ shifted to 1731.7 cm^−1^, demonstrating formation of a hydrogen bond between the N-H group of ERL and the carbonyl group of SOL. In the spectra of ERL control and ERL MPs, shifting and splitting of the N-H vibration peak around 3247.1 cm^−1^ demonstrated the crystal transformation of ERL, in agreement with the observations in PXRD and SEM. In addition, the shift of the SOL carbonyl group vibration from 1729.9 cm^−1^ to 1743.4 cm^−1^ could be due to two possible events. Firstly, the shift could result from the ERL crystal form transformation. Secondly, it might be due to strong hydrogen-bonding interactions between ERL and SOL in ERL MPs. Combining the FT-IR results with the FBRM data, it is very likely that with the presence of hydrogen bonding interactions between SOL and ERL, the nucleation process of ERL crystals was largely inhibited, which inhibited drug crystallization.

### 3.7. Dissolution

**Figure 8:**
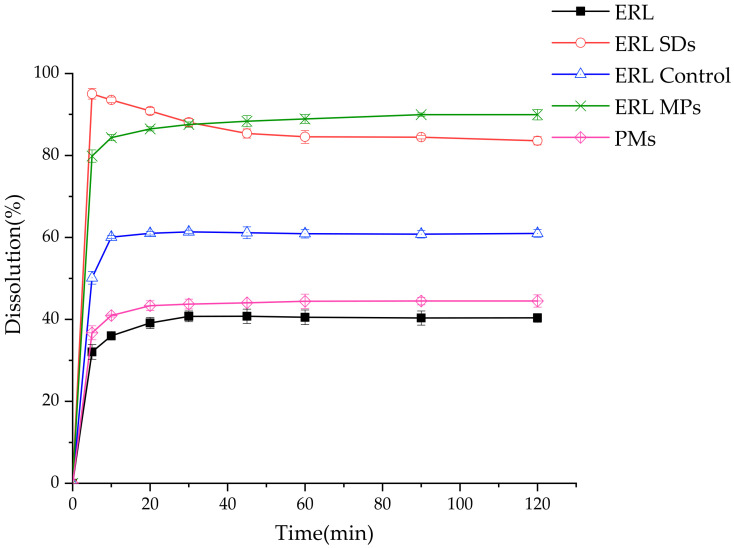
Dissolution curves of ERL, ERL SDs, ERL control, ERL MPs and PMs.

Figure 8 shows the dissolution curves for ERL, ERL SDs, ERL control, ERL MPs and PMs. Only 40% of ERL had dissolved after 120 min. The dissolution rate for the PM was slightly higher than that of pure ERL. This might be due to the solubilizing effect of SOL. The dissolution of ERL control was also increased, which could be attributed to the transformation of ERL crystal to metastable form A, the solubility of which is higher than that of stable crystal form B. Both the dissolution profiles of ERL SDs and ERL MPs were improved significantly via different potential mechanisms. Compared with the drug in the crystalline state, the amorphous drug is in a high energy state, which has a higher apparent solubility. Therefore, the largely elevated dissolution of ERL SDs could be due to its amorphous state. However, after 5 min, the dissolution of the SDs began to decrease gradually, which could be attributed to recrystallization of ERL driven by supersaturation. It should be noted that ERL released from ERL MPs reached as much as 90%. On one hand, the small particle size generated increased surface area; on the other hand, the solubility of metastable form A was higher than that of form B. In particular, the dissolution of ERL MPs was much higher than that of ERL Control. This could be explained by the hydrogen-bonding interactions between ERL and SOL observed by FT-IR. In addition, unlike ERL SDs, recrystallization did not occur in ERL MPs. This could be because the energy of metastable crystal form A was lower than that of the amorphous state.

### 3.8. Accelerated Stability Test

**Figure 9:**
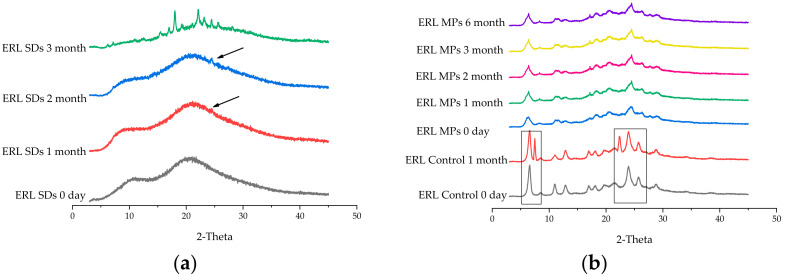
PXRD patterns of (**a**) ERL SDs and (**b**) ERL MPs stored at 40 °C/75% RH.

As shown in Figure 9a, a small diffraction peak was observed in the PXRD of ERL SDs after one month of storage. The size of this peak was increased after two months of storage; after three months, numerous peaks were visible. As shown in Figure 9b, characteristic peaks of form B were detected in ERL Control after one month of storage, indicating that form A of ERL was not sufficiently stable in ERL control. However, after six months, the results of PXRD showed that the peak did not change, indicating that there was no crystal transformation in ERL MPs, and the metastable form A was stable with the presence of SOL. In addition, the drug content and dissolution of ERL MPs stored for six months were determined, and there was no significant change compared with 0 days. According to SEM and FBRM observations, SOL exhibited a strong crystallization inhibitory effect on ERL. A small amount of SOL in ERL MPs enabled good stability of the metastable polymorph during storage. However, ERL SDs, which contained more than two times the amount of SOL, did not show good stability, indicating that the SDs had a higher lattice energy compared with MPs.

According to studies by Janssens S. and Van den Mooter G. [37], hydrogen bond formation between a drug and polymer could disrupt hydrogen bonds formed between drug molecules, which disfavors drug crystallization. Moreover, hydrogen bond formation between drug and polymer could limit diffusion of drug molecules, thus decreasing molecular mobility and inhibiting crystallization [38]. Here, hydrogen bond formation between ERL and SOL was observed by FT-IR, yet the SDs formed by ERL and SOL were not correspondingly stable. In contrast, MPs formed by ERL and SOL exhibited excellent stability.

The stable crystalline form of the drug has the lowest lattice energy and, at the same time, comparatively low dissolution. Usually, an amorphous form is preferred because of its superior solubility properties. However, the high energy amorphous form was thermodynamically unstable and can easily transform into the stable crystalline form during storage [39]. The metastable crystalline form is energetically between the stable crystalline and unstable amorphous forms, with lattice energy lower than that of the amorphous form but apparent solubility higher than that of the stable crystalline form [40]. In this study, addition of SOL during anti-solvent recrystallization stabilized the metastable crystal form A of ERL, which exhibited higher dissolution than the stable crystalline form and better stability than the amorphous state.

## 4. Conclusions

The widely used strategy of stabilization using SDs and MPs is useful for some drugs. However, a targeted and tailored strategy should be developed and applied for a specific drug. SOL is an amphiphilic polymer that has been shown to be effective for crystallization inhibition of the model drug ERL by FBRM. ERL SDs and MPs prepared in the presence of SOL exhibited significantly enhanced dissolution. For ERL SDs, the enhanced dissolution was mainly due to amorphous drug. For ERL MPs, the enhanced dissolution was primarily due to reduction of particle size and transformation of stable crystal form B to metastable form A, which had higher apparent solubility. ERL SDs did not show a good stability as expected, while ERL MPs exhibited long-term stability as crystal form A. On one hand, this may be because the amorphous form of ERL has higher energy and has a greater tendency to transform into stable crystalline form compared with the metastable crystalline form. On the other hand, SOL has strong hygroscopicity and water can increase molecular mobility to promote drug crystallization. According to studies by Andres Lust et al., Soluplus can inhibit drug crystallization at low humidity [18]. Plasticity occurs if the solid dispersion absorbs plenty of water. According to the Fox equation, the Tg of solid dispersion will decrease by about 10 °C for every 1% of water absorbed, which will enhance the molecular mobility of the drug and promote the crystallization [41]. While SOL strongly inhibited crystallization of ERL in solution, the inhibitory efficacies exhibited in SDs and MPs were different. Strategies should be tailored for a specific drug in order to get an appropriate approach. In addition, the results suggested that SOL, commonly used in SDs, could be used as a stabilizer in ERL MPs. Furthermore, the molecular interactions between drug and polymer can act as an indicator for stabilizer screening in pharmaceutical formulations. This study may provide new insights into the stabilization of amorphous and metastable states of drugs.

## Data Availability

The data presented in this study are available upon request to the corresponding author.

## References

[1] Williams H.D., Trevaskis N.L., Charman S.A., Shanker R.M., Charman W.N., Pouton C.W., Porter C.J. (2013). Strategies to sddress low drug solubility in discovery and development. Pharmacol. Rev..

[2] Chaudhari S.P., Dugar R.P. (2017). Application of surfactants in solid dispersion technology for improving solubility of poorly water soluble drugs. J. Drug Deliv. Sci. Technol..

[3] Siafaka P.I., Cağlar E.Ş., Papadopoulou K., Tsanaktsis V., Karantas I.D., Okur N.Ü., Karasulu H.Y. (2019). Polymeric microparticles as alternative carriers for antidiabetic glibenclamide drug. Pharm. Biomed. Res..

[4] Sahoo N.G., Kakran M., Li L., Judeh Z. (2010). Fabrication of composite microparticles of artemisinin for dissolution enhancement. Powder Technol..

[5] Sharma D., Sharma A., Aggarwal S., Sharma R.B. (2021). Different methods used in solid dispersion—A review. Int. J. Biol. Pharm. Allied Sci..

[6] Kanaujia P., Poovizhi P., Ng W.K., Tan R.B.H. (2015). Amorphous formulations for dissolution and bioavailability enhancement of poorly soluble APIs. Powder Technol..

[7] Moseson D.E., Corum I.D., Lust A., Altman K.J., Hiew T.N., Eren A., Nagy Z.K., Taylor L.S. (2021). Amorphous solid dispersions containing residual crystallinity: Competition between dissolution and matrix crystallization. AAPS J..

[8] Guan J., Huan X., Liu Q., Jin L., Wu H., Zhang X., Mao S. (2019). Synergetic effect of nucleation and crystal growth inhibitor on in vitro-in vivo performance of supersaturable lacidipine solid dispersion. Int. J. Pharm..

[9] Fan W., Xu Y., Zhang X., Di L., Li L. (2020). Study on solubility improvement of lidocaine by ternary solid dispersion. J. Chin. Pharm. Sci..

[10] Bharate S.S. (2021). Enhancing biopharmaceutical attributes of khellin by amorphous binary solid dispersions. AAPS PharmSciTech.

[11] Li J., Wang Z., Zhang H., Gao J., Zheng A. (2021). Progress in the development of stabilization strategies for nanocrystal preparations. Drug Deliv..

[12] Keck C.M., Muller R.H. (2006). Drug nanocrystals of poorly soluble drugs produced by high pressure homogenization. Eur. J. Pharm. Biopharm..

[13] Uhlemann J., Diedam H., Hoheisel W., Schikarski T., Peukert W. (2021). Modeling and simulation of process technology for nanoparticulate drug formulations—A particle technology perspective. Pharmaceutics.

[14] Mahesh K.V., Singh S.K., Gulati M. (2014). A comparative study of top-down and bottom-up approaches for the preparation of nanosuspensions of glipizide. Powder Technol..

[15] Dong Y., Ng W.K., Shen S., Kim S., Tan R.B. (2011). Controlled antisolvent precipitation of spironolactone nanoparticles by impingement mixing. Int. J. Pharm..

[16] Kumar R., Dalvi S.V., Siril P.F. (2020). Nanoparticle-based drugs and formulations: Current status and emerging applications. ACS Appl. Nano Mater.

[17] Schram C.J., Beaudoin S.P., Taylor L.S. (2016). Polymer inhibition of crystal growth by surface poisoning. Cryst. Growth Des..

[18] Lust A., Strachan C.J., Veski P., Aaltonen J., Heinämäki J., Yliruusi J., Kogermann K. (2015). Amorphous solid dispersions of piroxicam and Soluplus: Qualitative and quantitative analysis of piroxicam recrystallization during storage. Int. J. Pharm..

[19] Xie T., Taylor L.S. (2016). Dissolution performance of high drug loading celecoxib amorphous solid dispersions formulated with polymer combinations. Pharm. Res..

[20] Shi N.Q., Lei Y.S., Song L.M., Yao J., Zhang X.B., Wang X.L. (2013). Impact of amorphous and semicrystalline polymers on the dissolution and crystallization inhibition of pioglitazone solid dispersions. Powder Technol..

[21] Chen N., Di P., Ning S., Jiang W., Jing Q., Ren G., Liu Y., Tang Y., Xu Z., Liu G. (2019). Modified rivaroxaban microparticles for solid state properties improvement based on drug-protein/polymer supramolecular interactions. Powder Technol..

[22] Linn M., Collnot E.M., Djuric D., Hempel K., Fabian E., Kolter K., Lehr C.M. (2012). Soluplus as an effective absorption enhancer of poorly soluble drugs in vitro and in vivo. Eur. J. Pharm. Sci..

[23] Nandi U., Ajiboye A.L., Patel P., Douroumis D., Trivedi V. (2021). Preparation of solid dispersions of simvastatin and soluplus using a single-step organic solvent-free supercritical fluid process for the drug solubility and dissolution rate enhancement. Pharmaceuticals.

[24] Shamma R.N., Basha M. (2013). Soluplus: A novel polymeric solubilizer for optimization of Carvedilol solid dispersions: Formulation design and effect of method of preparation. Powder Technol..

[25] Cohen M.H., Johnson J.R., Chen Y.F., Sridhara R., Pazdur R. (2005). FDA drug approval summary: Erlotinib (Tarceva) Tablets. Oncologist.

[26] Dora C.P., Kushwah V., Katiyar S.S., Kumar P., Pillay V., Suresh S., Jain S. (2017). Improved oral bioavailability and therapeutic efficacy of erlotinib through molecular complexation with phospholipid. Int. J. Pharm..

[27] Sodhi I., Mallepogu P., Thorat V.P., Kashyap M.C., Sangamwar A.T. (2019). Insights on role of polymers in precipitation of celecoxib from supersaturated solutions as assessed by focused beam reflectance measurement (FBRM). Eur. J. Pharm. Sci..

[28] Chauhan H., Kuldipkumar A., Barder T., Medek A., Gu C.H., Atef E. (2014). Correlation of inhibitory effects of polymers on Indomethacin precipitation in solution and amorphous solid crystallization based on molecular interaction. Pharm. Res..

[29] Sanphui P., Rajput L., Gopi S.P., Desiraju G.R. (2016). New multi-component solid forms of anti-cancer drug Erlotinib: Role of auxiliary interactions in determining a preferred conformation. Acta Crystallogr. Sect. B.

[30] Tien Y.C., Su C.S., Lien L.H., Chen Y.P. (2010). Recrystallization of erlotinib hydrochloride and fulvestrant using supercritical antisolvent process. J. Supercrit. Fluids.

[31] Wang H., Zhang X., Zhang P., Yang Y., Yuan Z., Yu X. (2009). Polymorph form L of erlotinib, methods of preparation and uses thereof.

[32] Thorat S.H., George C.P., Shaligram P.S., Suresha P.R., Gonnade R.G. (2021). Polymorphs and hydrates of the anticancer drug erlotinib: X-ray crystallography, phase transition and biopharmaceutical studies. CrystEngComm.

[33] Song Y., Yang X., Chen X., Nie H., Byrn S., Lubach J.W. (2015). Investigation of drug-excipient interactions in Lapatinib amorphous solid dispersions using solid-state NMR spectroscopy. Mol. Pharm..

[34] Perera M.D., Sinha A.S., Aakeröy C.B. (2018). Using structural mimics for accessing and exploring structural landscapes of poorly soluble molecular solids. Acta Crystallogr. Sect. B Struct. Sci. Cryst. Eng. Mater..

[35] Penumetcha S.S., Gutta L.N., Dhanala H., Yamili S., Challa S., Rudraraju S., Rudraraju S., Rudraraju V. (2016). Hot melt extruded aprepitant-soluplus solid dispersion: Preformulation considerations, stability and in vitro study. Drug Dev. Ind. Pharm..

[36] Karavas E., Koutris E., Papadopoulos A.G., Sigalas M.P., Nanaki S., Papageorgiou G.Z., Achilias D.Z., Bikiaris D.N. (2014). Application of density functional theory in combination with FTIR and DSC to characterise polymer drug interactions for the preparation of sustained release formulations between fluvastatin and carrageenans. Int. J. Pharm..

[37] Janssens S., Van den Mooter G. (2009). Review: Physical chemistry of solid dispersions. J. Pharm. Pharmacol..

[38] Bhugra C., Pikal M.J. (2008). Role of thermodynamic, molecular, and kinetic factors in crystallization from the amorphous state. J. Pharm. Sci..

[39] Tian B., Ju X., Yang D., Kong Y., Tang X. (2020). Effect of the third component on the aging and crystallization of cinnarizine-soluplus binary solid dispersion. Int. J. Pharm..

[40] Censi R., Di Martino P. (2015). Polymorph impact on the bioavailability and stability of poorly soluble drugs. Molecules.

[41] Leuner C., Dressman J. (2000). Improving drug solubility for oral delivery using solid dispersions. Eur. J. Pharm. Biopharm..

